# Improving shared decision-making in advanced Parkinson’s disease: protocol of a mixed methods feasibility study

**DOI:** 10.1186/s40814-018-0286-4

**Published:** 2018-07-04

**Authors:** Frouke A. P. Nijhuis, Glyn Elwyn, Bastiaan R. Bloem, Bart Post, Marjan J. Faber

**Affiliations:** 1 Department of Neurology, Canisius Wilhelmina Hospital, Nijmegen, the Netherlands; 2 Department of Neurology, Radboud Institute for Health Sciences, Radboud University Medical Center, Neurology 935, PO Box 9101, 6500 HB Nijmegen, the Netherlands; 3 The Dartmouth Institute for Health Policy and Clinical Practice, Lebanon, NH USA; 4 Department of Neurology, Radboud University Medical Center, Donders Institute for Brain, Cognition and Behaviour, Nijmegen, the Netherlands; 5 Radboud Institute for Health Sciences, Radboud University Medical Center, Nijmegen, the Netherlands

**Keywords:** Protocol, Feasibility study, Shared decision-making, Parkinson’s disease, Decision aid, Mixed methods design

## Abstract

**Background:**

In advanced stages of Parkinson’s disease (PD), patients and neurologists regularly face complex treatment decisions. Shared decision-making (SDM) can support the process where evidence, the clinician’s expertise and the patient’s preferences jointly contribute to reach an optimal decision. Here, we describe the rationale of our feasibility study protocol.

The aim of the study is to test the feasibility of the SDM intervention by (1) analysing the acceptability of the intervention by users (i.e. professionals and patients), (2) assessing the level of implementation, (3) testing efficacy on a small scale and (4) evaluating the study procedures.

**Methods:**

Using an uncontrolled before-after mixed methods design, patients in the pre-intervention group will receive information and decisional support as usual. Patients in the post-intervention group will receive the SDM intervention, consisting of an Option Grid™ patient decision aid and a website with supplementary information plus a value clarification tool for both patients and professionals. An Option Grid is a one-page, evidence-based summary of available options, listing the frequently asked questions that patients consider when making treatment decisions. A value clarification tool helps patients identify which option he/she prefers based on attributes in the treatment decision context. Neurologists and PD nurse specialists will receive a 1-h instruction on SDM and how to use the SDM intervention.

Through purposive sampling, neurologists and PD nurse specialists will be recruited from both specialised neurology clinics and community-based hospitals. Included professionals will invite consecutive patients who are eligible for the advanced therapies.

Data will be collected using questionnaires, interviews and audio observations of the consultations and by tracking users’ logging behaviour of the website. Data will be analysed using a mixed methods design.

**Discussion:**

The mixed methods design will create a deeper understanding of how the SDM intervention affects the interactions between professionals (a neurologist and/or a PD nurse specialist) and the patient, when an advanced treatment is chosen. The results of the study will inform the design of an RCT to test the effectiveness of the SDM intervention.

**Trial registration:**

NTR6649, retrospectively registered 28 August 2017.

## Background

In the advanced stage of Parkinson’s disease (PD), disability accumulates significantly due to a range of motor and non-motor complications. Of these, motor fluctuations have a major impact on quality of life and it has been shown that advanced treatments (deep brain stimulation (DBS), levodopa-carbidopa intestinal gel (LCIG) or continuous subcutaneous apomorphine infusion (CSAI)) improve motor function and may thus convey substantial benefit. However, neurologists and PD patients face a complex decision choosing between available treatment options and selecting the one that best suits the patient’s needs. For various reasons, the application of the evidence-based medicine principles to this particular decision is challenging [1]. Randomised controlled trials comparing all three advanced treatments are lacking [2], and many professionals fail in bridging this knowledge gap due to a lack of treatment experience or availability at their site [3]. Consequently, a large proportion of PD patients are not fully informed on all advanced treatment options by their neurologists [3–5]. And more importantly, PD patients feel insufficiently involved in the decision-making process [3, 6].

To overcome the aforementioned limitations in the decision-making process, shared decision-making (SDM) can play a pivotal role [7]. SDM represents the process of decision-making in which the professional and patient, and possible other key players, define the decision to be made, share the treatment options and available evidence, elicit the patient’s preferences and reach a shared decision [8–10]. SDM can be facilitated by a decision aid [10]. A decision aid states the decision to be made on a health problem, provides the available evidence on the options, including the scientific uncertainties, and supports patients in clarifying what values are important to them personally. A decision aid thus supports the patients and professionals to actively participate in decision-making [11].

In this paper, we describe the design of a feasibility study examining the SDM intervention in the decision-making process for an advanced therapy in PD. The intervention we designed consists of an Option Grid ™ patient decision aid, online supplementary information with a value clarification tool and a 1-h training for professionals on SDM and how to use the SDM intervention. An Option Grid is a one-page, evidence-based summary of available options presented in a tabulated format, listing the frequently asked questions that patients consider when making treatment decisions [12]. An Option Grid is used in the clinical encounter and stimulates the discussion about the treatment options [13]. A value clarification tool helps a patient to think about which attributes of options matter to him/her most, to identify which option fits best his/her personal life [14].

The study will address the feasibility of the SDM intervention by (1) analysing the acceptability of the intervention by users (i.e. professionals and patients), (2) assessing the level of implementation, (3) testing efficacy on a small scale and (4) evaluating the study procedures.

## Methods

### Study design

This feasibility study has a multi-centre, uncontrolled before-after, mixed methods design, with five participating centres. A before-after design creates the possibility for professionals to reflect on their practice pre- and post-intervention. The rationale for a feasibility study is to first examine if the intervention will be accepted by its users and can be implemented in clinical practice before we can test effectiveness [15]. The ideal sample size for a feasibility study ranges between 24 and 50 patients for the total sample size [16, 17]. We aim for a total of 40 patients at this stage: 20 in the pre-intervention group (baseline group) and 20 in the post-intervention group. Based on our previous study [4], we estimate that the majority of professionals in community-based hospitals consider three to ten patients for an advanced therapy per year. Because the SDM intervention is a complex intervention, using a mixed methods design will achieve cross-validation or triangulation of the data. With the triangulation, we aim to gain a more complete understanding of the effects of the SDM intervention [18]. To increase the understanding, we will also measure contextual factors, such as cognitive deficits in patients and treatment expertise of professionals that can influence the outcome measures.

### Participants

#### Hospitals

Five hospitals in the Netherlands will participate and were selected from our previous studies [3, 4]. Specialised neurology centres and community-based hospitals will be represented, with variable accessibility to advanced PD treatments.

#### Professionals

Neurologists will be eligible if they (1) consider at least five PD patients per year for advanced treatment and (2) collaborate with a PD nurse specialist in the same hospital. We aim for a total of five to ten neurologists. A participating hospital can provide more than one neurologist. Moreover, we aim to include professionals with different levels of expertise on the advanced treatment options.

#### Patients

Patients are included in a convenient consecutive sample and can be included if they (1) are diagnosed with advanced PD and are considered to be a suitable candidate for advanced treatment, judged by their own neurologist and (2) are eligible for all three treatments at the beginning of the decision-making process. Patients who currently have or previously underwent an advanced treatment for PD are excluded.

### The intervention

The intervention has been developed following the process map for web-based decision support interventions [19]. The SDM intervention consists of an Option Grid patient decision aid and a website with supplementary information plus a value clarification tool, and a 1-h training for professionals. The key element of the SDM intervention is the Option Grid, and it will be used in the first encounter to start the discussion on the treatment options. Patients will then be referred to the website for more elaborate information. The online supplementary information is divided into a patient-dedicated site and a professional-dedicated site. The patient site provides a short instruction video about the website, more detailed information on the decision to be made, the Option Grid itself, detailed information on the treatment options and the value clarification tool. The professional site presents the Option Grid, a template for the decision process and the evidence synthesis on which the Option Grid is based. Both sites are transparently accessible for both patients and professionals.

The main researcher (FN) provides an on-site 1-h interactive training to the participating neurologists and PD nurse specialists. During the training, the concept of SDM and the deliberative consultation model will be introduced [8]. Furthermore, the use of the Option Grid patient decision aid and the online supplementary information will be explained. The training will not be extensive as we want to analyse how professionals use the SDM intervention in real-life situations, without any prescribed behaviour, as the Option Grid patient decision aid will be publicly available without training after the study [12].

### Study procedures

Consecutive patients, found to be eligible for an advanced treatment by the neurologist, will be invited to participate in the study. The patients will receive written information from their neurologist or PD nurse specialist, before written informed consent is obtained.

In the pre-intervention group, patients will receive information and decision support as usual. The decision process of an individual patient ends when a preliminary choice for a treatment has been made and the screening for definite treatment eligibility is initiated.

Once 20 patients in the pre-intervention group have finished their decision process, the professionals will participate in the 1-h training. Next, the 20 patients of the post-intervention group will be included and enter the decision process making use of the Option Grid patient decision aid and website. Every neurologist and PD nurse specialist will receive a personal login code after the training for the website. Once patients in the post-intervention group are included and have been introduced to the Option Grid by the professional, they will receive their unique login code as well.

### Outcome measures

This feasibility study will focus on four aspects, being acceptability, level of implementation, small-scale efficacy testing and evaluation of study procedures, each with their own relevant outcome measures (Table 1). In order to increase cross-validation of the data contextual factors will also be measured (Table 1).

**Table 1:** Study methods and instruments

Research objectives	Components	Methods and instruments
Demographic data	Demographic data	Baseline questionnaire with questions on demographics
Acceptability	1. Satisfaction with intervention and how intervention is received	1a. Structured questionnaires for patients on items as readability, comprehensiveness, layout and amount of information 1b. Semi-structured interviews with patients, neurologists and PD nurse specialists with items on perceived satisfaction, and perceived strengths and weaknesses of the intervention
Level of implementation	1. To what extent is the intervention implemented as planned 2. To what extent were all components of the intervention used 3. How did participants react to the specific aspects of the intervention and to what extent did patients engage in the intervention 4. What proportion of the included population actually were using the intervention	1. Field notes to what extent the intervention was implemented as planned and training was provided as planned 2. Analysis of audiotapes consultations, logging data of navigation behaviour website and hard copies of value elicitation tool summary: evaluation if all elements of intervention are actually used 3a. Analysis of audiotapes consultations and logging data of navigation behaviour website to analyse if patients engage in all elements of the intervention 3b. Semi-structured interviews on the perceived interaction with the different elements of the intervention 4. Analysis of audiotapes consultations and logging data of navigation behaviour website to analyse which patients in the intervention group and professionals were using the intervention during the decision process
Small-scale efficacy testing: - Level of SDM	1. Patient and neurologist/PD nurse perceived level of SDM 2. Researcher observed level of SDM	1. Structured questionnaires for patients and neurologists/PD nurse specialists using the following validated scales: SDMQ-9 (patients) and SDMQ-9-doc (neurologists/PD nurse specialists), CollaboRATE (patients) and CPS actual role (patients, neurologists, PD nurse specialists) 2. Analysis of the audiotaped consultations using the validated scale: OPTION-5
Small-scale efficacy testing:- Decision quality	1. Level of informed choice 2. Decisional conflict in decision-making	1. Measuring knowledge in patients at the start and end of decision-making process using questionnaire with 20 questions on the advanced treatments 2. Structured questionnaires for patients and neurologists/PD nurse specialists using the following validated scales: DCS (patients) and PDPAI (neurologists/PD nurse specialists)
Feasibility of study procedures	1. Recruitment 2. Potential outcome indicators 3. Approaches to data collection	1a. Field notes on study inclusion rate, drop-out rates 1b. Semi-structured interviews with neurologists and PD nurse specialists with items such as barriers to recruitment 2. Analysis of outcome measures from the small-scale efficacy testing with evaluation of conflicting data on outcome measures 3. Semi-structured interviews with patients and neurologists/PD nurse specialists with items on acceptability of the logistics/practicability of the study procedures
Context	1. Patient-related factors in the implementation and outcomes 2. Professional-related factors in the implementation and outcomes 3. Organisational context	1a. Structured questionnaires for patients with items on preferred role in decision-making (CPS), treatment preference, pre-knowledge, health literacy skills (FCHHL) and mood (HADS) 1b. Cognitive testing using MoCA, BSAT, Verbal Fluency, Stroop Color Word Test, National Adult Reading Test, Raven Advanced Progressive Matrices and MMSE 2. Structured questionnaires for neurologists and PD nurse specialists with items on their role in decision-making (CPS), treatment preference and level of experience with treatments 3. Field notes on national consensus of treatment of advanced PD, organisational structure for this specific decision in participating centres

*CPS* control Preference Scale, *FCCHL* Functional Communicative and Critical Health Literacy, *HADS* Hospital Anxiety and Depression Scale, *MoCA* Montreal Cognitive Assessment, *BSAT* Brixton Spatial Anticipation Test Provider Decision Process Assessment Instrument, *MMSE* Mini Mental State Examination, *PDPAI* Provider Decision Process Assessment Instrument

#### Acceptability of the SDM intervention

Acceptability evaluates how the neurologists, PD nurse specialists and patients react to the intervention [20]. Assessment of acceptability is paramount as it predicts whether the intervention will be adopted and used. Acceptability will be evaluated based on a manual for testing acceptability of a decision aid [21]. Acceptability is tested by questions on readability, comprehensiveness, layout, amount of information as well as the professional’s and patient’s opinions about the SDM intervention and suggestions for improvement.

#### Level of implementation of the SDM intervention

Implementation evaluates whether the SDM intervention is executed as planned. The level of implementation will be evaluated in terms of the changes made to the SDM intervention while using it, the perceived level of implementation of the SDM intervention, and the experienced barriers and facilitators in using the intervention. Moreover, the utilisation of the different elements of the SDM intervention will be measured.

#### Small-scale efficacy testing of the SDM intervention

The aim of the intervention is to improve SDM and the quality of the decision in advanced PD. Testing the feasibility of the intervention also includes a small-scale efficacy testing to guide the choice of outcome measures for the future RCT [21]. We will focus on two efficacy outcome domains. First, the elements within the SDM process that are used during the consultations (‘level of SDM’) are assessed in a triadic approach, meaning that the level of SDM is evaluated from three perspectives (i.e. patient, professional, observer). It includes four validated measurement instruments, i.e. OPTION-5 [22] (observational measurement), CollaboRATE [23], SDMQ9-patient (patient-reported outcome measures) and SDMQ9-doc (professional-reported outcome measure) [24].

Secondly, we assess the quality of decision-making. A decision is considered of high quality if it is based on sufficient knowledge and is in alignment with one’s personal preferences [25]. To capture this, we have selected three instruments, i.e. the level of informed choice (knowledge test), the Decisional Conflict Scale (DCS) [26] and the Provider Decision Process Assessment Instrument (PDPAI) [27, 28].

#### Feasibility of study procedures

An evaluation of the study procedures is performed to learn lessons for a future randomised controlled trial. Inclusion procedures and drop-out rates will be monitored. The interviews will address the professional’s and subject’s conflicting responses on outcome measures, opinions about the study procedures and suggestions for improvement. Field notes will be used to evaluate any logistical problems in the study procedures.

#### Context

In order to correctly interpret the results on the abovementioned outcomes, it is imperative to know which external factors influence the acceptability, implementation and outcomes [29–31]. We have defined a limited set of contextual factors, based on our previous findings [3]. They include (1) cognitive deficits in PD patients that could limit the use of the SDM intervention, (2) treatment preference of patients at forehand that could influence which components of the SDM intervention are used, (3) level of experience with the treatments of the professionals that could influence how they introduce the SDM intervention to the patients and (4) organisational factors such as changing consensus nationally on advanced treatments.

### Data collection

#### Cognitive testing

The main researcher (FN) will visit the patient at his/her home to conduct the cognitive test battery. The estimated time to complete the cognitive test battery is around 45 min. The researcher will try to conduct the test at a moment after the patient has taken medication to ensure optimal testing conditions. However, patients in this stage of the disease can have unpredictable fluctuations in functioning during the day. The test battery will be conducted in the same order in each patient to eliminate changes between different tests due to fatigue or loss of concentration.

#### Questionnaires

Professionals will complete a baseline questionnaire at the start of the study to collect demographic information and an exit questionnaire to collect their experiences with the SDM intervention at the end of the study. They will also complete two short digital questionnaires per patient: one at the start and one at the end of the decision-making process. The two short questionnaires will contain the outcome measures for small-scale efficacy testing.

Patients will complete a baseline questionnaire to collect demographic information immediately after inclusion. After the first consultation when the options have been discussed, patients will be invited to complete a questionnaire which contains questions about contextual factors that could interact with the decision-making process and some of the outcome measures for the efficacy testing. The next questionnaire will be sent immediately after a decision for a treatment has taken place, and this questionnaire will address outcome measures for efficacy testing. Finally, patients will receive an exit questionnaire to collect feedback on the decision process. In the post-intervention group, this questionnaire also addresses the SDM intervention.

#### Observations

We aim to audiotape all consultations covering the decision-making process, i.e. two to three per patient. It may prove difficult to record the first consultation when the professional introduces the option to change treatment, as this can occur before informed consent has been obtained. Consent for the audiotapes will be included explicitly in the consent form.

#### Interviews

Interviews with professionals will be conducted at the end of the study. The interviews will cover in-depth analysis on the acceptability of the intervention, how the intervention was used and barriers and facilitators for the integration into the work process, suggestions for improvement, as well as feasibility of the study procedures.

Interviews will be conducted with all patients at the end of their decision-making process. The interviews will elaborate on the decision process and feasibility of study procedures. Patients in the post-intervention group will also be asked how the SDM intervention was used in the decision process, the acceptability of the intervention, barriers and facilitators to implementation and suggestions for improvement.

#### Tracking navigation behaviour of the website

Tracking the online navigation behaviour will only be applied in the post-intervention part of the study protocol. The quantitative measures of utilisation of the SDM intervention website will be obtained through tracking analysis software, capturing both time-dependent (duration of website and component use) and navigation-dependent (navigational route, navigational step count, print commands, use of dedicated sites) variables. Patients will be asked to hand in the printed summary of the value clarification exercise as a source of information to see if patients use the value clarification tool.

#### Field notes

Field notes by the researcher are used to evaluate any difficulties observed in the study procedures, such as inclusion rate, difficulties using the SDM intervention, and problems filling in the questionnaires. It is possible that the SDM intervention has to be adapted during the study. Adaptations can be necessary to make the intervention fit into different contexts. These adaptations will be reported in field notes, taken by the researchers throughout the study period. External factors that can influence the outcome measures and are observed during the study period will also be written down by the researcher in the field notes. An example is for instance if the national consensus changes on what appropriate advanced treatment options are, which would change the options that have to be included in the Option Grid and website (Table 2).

**Table 2:**
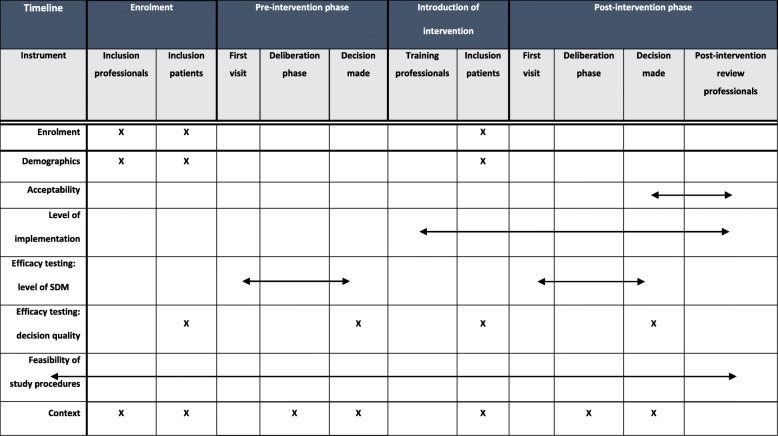
Data collection scheme

### Sample size calculation

As this is a feasibility study, we have not performed a sample size calculation [32]. The rationale for the sample size of 20 pre- and 20 post-intervention group participants has been given above in the study design section.

### Data analysis

For multidimensional understanding of the “black box of decision-making” and increasing validity of the data, we will create a mixed methods matrix combining the quantitative and qualitative data and use a triangulation protocol [33, 34].

#### Quantitative analysis

Descriptive statistical analysis will be conducted on sociodemographic characteristics of patients and professionals. To assess the feasibility of this study, descriptive analyses will be conducted on: inclusion rate (number of participant inclusions per quarter), retention rate (number of participants completing full study procedures), intervention implementation rate (number of participants in post-intervention group using intervention) and missing data (proportion fully completed per scale/questionnaire). Quantitative data will be reported as percentages, numeric counts, mean ± standard deviation (SD) or median and interquartile range (IQR), as appropriate. Differences between the pre- and post-intervention values will be tested parametrically or non-parametrically, depending on the outcomes being normally or not normally distributed. A *p* value less than 0.05 will be considered statistically significant. All results will be presented with 95% confidence intervals. Any hypothesis testing will be treated as preliminary and the results interpreted with caution as the study is likely to be underpowered to detect statistically significant differences. Unintended or unexpected effects will be documented. Results of any other analyses performed, which could be used to inform the future definitive RCT, will be explicitly described [35]. All analyses will be conducted using SPSS (Statistical Package for Social Sciences, Chicago, IL, USA).

#### Qualitative analysis

Audio-recordings of the consultations and the interviews will be transcribed verbatim. We will qualitatively analyse all consultations and interviews using thematic analysis [36]. Themes or patterns within data can be identified in an inductive or ‘bottom up’ manner, or in a theoretical or deductive, ‘top down’ manner [36]. As the analysis will be based on our previous work [3] and other literature, the theoretical analysis is more appropriate. Thematic analysis will consist of a cycle of several steps. In the initial phase, two researchers will familiarise themselves with the data by reading through all transcripts several times and generate initial codes. A code is any interesting feature, which relates to the main research question, mentioned by the participants (patients and/or professionals) in the interviews or an outstanding observation from the consultations. We will cluster the codes into categories, when they describe or relate to the same phenomena. In the next phase, codes can be reassigned or renamed, after which themes are developed. Next, the categories and themes will be discussed with the research team and codes and categories will be reevaluated. The final step is to interpret the data and reach consensus on the categories and main themes [36]. Atlas-ti will be used to support the qualitative analysis. We will follow the recommendations outlined in the COREQ criteria as much as possible to report the qualitative data [37].

#### Mixed methods analysis

The aim of the triangulation is to explore how the SDM intervention actually influences the decision process, creating a multidimensional understanding [38]. Exploring the mechanisms of impact through which the SDM intervention works helps in understanding the outcome results and can help identify which adaptations to the intervention are necessary to change unwanted effects. The mechanisms of impact will be analysed using triangulation [31].

We will apply two types of triangulation: data triangulation and methodological triangulation. The data triangulation means that data from neurologists, PD nurse specialists and patients will be used. The methodological triangulation is represented by different methods of data collection, using questionnaires, observations, interviews and field notes and by tracking logging behaviour. For the analyses, we will first sort the data and then apply a convergence coding scheme, analyse the level of agreement or dissonance by convergence assessment and evaluate complementarity and divergence. Complementarity analyses explore whether different findings together explain a phenomenon or outcome and divergence reflects a disagreement between results from different sources. Each of these contributes to enhance the validity of the research findings [34]. After analysing all data, results will be presented to the research group (all authors). Based on a group discussion, the researchers will work towards an agreement on the findings and their interpretation [33].

## Discussion

The study will address the following questions: (1) is the intervention accepted in its current state for implementation, (2) can the intervention be delivered as intended and (3) what are the most useful outcome measures to evaluate the impact of the intervention in a RCT? Possible problems that we anticipate on are the following:

- Difficulty with inclusion rate: As mentioned before, each neurologist has a limited number of patients eligible for these advanced treatments. The patient has to be eligible for all three treatments, and this could reduce the actual number of eligible patients per professional, which could limit inclusion rate.
- Difficulty with obtaining all outcome measures: As this specific decision-making process is complex and involves multiple consultations with the patient, neurologist and PD nurse specialist. This increases the risk of missing data, especially of the audiotapes. We will try to limit this risk by providing both the individual patient and the professionals with a voice recorder. However, we cannot prevent that the professional has already explained a bit on the three options before the patient is included, and therefore, a part of the decision-making process is missing on the audiotapes.
- As it is a convenient consecutive sample of patients, differences in baseline demographics can influence the outcome measures.
- Professionals included in the study know we will introduce a SDM intervention that should improve SDM. They are asked to give care as usual in the pre-intervention group; however, it could be that they will change their behaviour because they are evaluated.

The results of this project will evaluate the feasibility of the SDM intervention with all these constraints taken into consideration and will inform the design of a RCT in clinical practice to evaluate the intervention on effectiveness.

Our long-term goal is to offer patients the opportunity to actively participate in the self-management of their disease, including involvement in complex medical decision-making.

### Trial status

Patient inclusion started in May 2015. In April 2018, 75% of the total inclusion was completed and inclusion is ongoing.

## Abbreviations

CSAI: Continuous subcutaneous apomorphine infusion
DBS: Deep brain stimulation
LCIG: Levodopa-carbidopa intestinal gel
PD: Parkinson’s disease
RCT: Randomised controlled trial
SDM: Shared decision-making
